# C-857-02. Balancing Ethical Principles and Medical Futility in Burn Transfers

**DOI:** 10.1093/jbcr/irag033.102

**Published:** 2026-04-06

**Authors:** Alexander Rowan, Alexander Chen, Dustin Dillon, Samuel A Matthys, Rabia Nizamani

**Affiliations:** Leon S. Peters Burn Center, Las Vegas, NV; University of Nevada, Las Vegas, NV; University of Nevada Las Vegas, Department of General Surgery, Las Vegas, NV; Leon S. Peters Burn Center, Las Vegas, NV; University Medical Center Lions Burn Care Center, Las Vegas, NV

## Abstract

**Background:**

Critically ill patients who present to non-burn centers with likely non-survivable burns are often still transferred to burn centers, despite an extremely poor prognosis. Federal mandates such as EMTALA (Emergency Medical Treatment and Labor Act) are important to ensure access to specialized care for all, but do not provide guidance on the challenges of determining medical futility at a non-specialized center. These challenging situations force clinicians to reconcile the ethical principles of beneficence (acting in the patient’s best interest), non-maleficence (avoiding interventions that prolong suffering), and justice (fair allocation of finite critical care and burn resources).

**Methods:**

Case report and review of the ethics of medical futility and distributive justice.

**Results:**

The patient was an unidentified man initially transported to a community hospital without burn capability. Upon arrival, he was hyperthermic to 108.9°F with TBSA 30% pavement burns to the posterior torso and lower extremities, and GCS of 3. He was in multisystem organ failure with ventricular arrhythmias requiring cardioversion, an NSTEMI, acute kidney injury, and disseminated intravascular coagulation. Soon after transfer to the regional burn center, the patient became pulseless and required two rounds of ACLS to achieve return of spontaneous circulation. Despite ICU admission, multispecialty consultation, and maximal vasopressor support, the patient expired within 12 hours of transfer. Notably, he was never identified and no family was found to guide medical decisions. Therefore, he received maximal medical effort.

**Conclusions:**

When care is deemed futile, the ethical principles of non-maleficence and distributive justice suggest no further escalation of care. In the case of burn patients, consultation with a burn center can help clinicians determine futility prior to transfer. If transfer is considered, ensuring goals-of-care discussions are conducted is critical to informing appropriate clinical decision making. This case highlights the challenges physicians face when balancing caring for critically ill patients with attention to resource stewardship.

**Applicability to Practice:**

When there is a question of medical futility in patients needing specialized care, important considerations include goals of care discussions and close communication between providers directly caring for the patient and physicians at specialized centers.

